# Potential Plant-Based New Antiplasmodial Agent Used in Papua Island, Indonesia

**DOI:** 10.3390/plants12091813

**Published:** 2023-04-28

**Authors:** Raden Bayu Indradi, Muhaimin Muhaimin, Melisa Intan Barliana, Alfi Khatib

**Affiliations:** 1 Department of Biological Pharmacy, Faculty of Pharmacy, Universitas Padjadjaran, Sumedang 45363, Indonesia; 2 Center of Herbal Study, Universitas Padjadjaran, Sumedang 45363, Indonesia; 3 Center of Excellence in Pharmaceutical Care Innovation, Universitas Padjadjaran, Sumedang 45363, Indonesia; 4 Department of Pharmaceutical Chemistry, Kuliyyah of Pharmacy, International Islamic University Malaysia, Kuantan 25200, Malaysia

**Keywords:** antiplasmodial, medicinal plants, Papua Island

## Abstract

Resistance to antimalarial medicine remains a threat to the global effort for malaria eradication. The World Health Organization recently reported that artemisinin partial resistance, which was defined as delayed parasite clearance, was detected in Southeast Asia, particularly in the Greater Mekong subregion, and in Africa, particularly in Rwanda and Uganda. Therefore, the discovery of a potential new drug is important to overcome emerging drug resistance. Natural products have played an important role in drug development over the centuries, including the development of antimalarial drugs, with most of it influenced by traditional use. Recent research on traditional medicine used as an antimalarial treatment on Papua Island, Indonesia, reported that 72 plant species have been used as traditional medicine, with *Alstonia scholaris*, *Carica papaya*, *Andrographis paniculata*, and *Physalis minima* as the most frequently used medicinal plants. This review aimed to highlight the current research status of these plants for potential novel antiplasmodial development. In conclusion, *A. paniculata* has the highest potential to be developed as an antiplasmodial, and its extract and known bioactive isolate andrographolide posed strong activity both in vitro and in vivo. *A. scholaris* and *C. papaya* also have the potential to be further investigated as both have good potential for their antiplasmodial activities in vivo. However, *P. minima* is a less studied medicinal plant; nevertheless, it opens the opportunity to explore the potential of this plant.

## 1. Introduction

Currently, malaria remains the most severe parasitic disease globally. In 2019, the World Health Organization (WHO) estimated there had been 227 million cases and 558,000 deaths from malaria worldwide. In 2020, the number rose to approximately 241 million cases and 667,000 deaths after the COVID-19 pandemic disrupted services [1]. The reported global incidence rate reduced between 2000 and 2019. However, from 2015 to 2019, the change in the incidence slowed dramatically, and mortality rate reduction was reported to be slower in 2015–2019 [2]. The South-East Asia (SEA) region ranks second behind the African Region in the estimated number of malaria cases, and Indonesia is one of nine malaria-endemic countries in the SEA. The malaria elimination goal in Indonesia must be achieved by 2030 [2,3]. In 2021, the Indonesian Ministry of Health released the Indonesian Health Profile. The Annual Parasite Incidence (API) at the national level was at 0.94 per 1000 people. However, the Papua and Papua Barat provinces still record significantly higher numbers than national values, with 63.12 and 10.15 per 1000 people, respectively, and 95–99% received artemisinin combination treatment (ACT) [4].

Resistance to antimalarial medicine remains a threat to the global effort in malaria eradication. On the way to eliminate malaria, resistance to several drugs such as quinine, chloroquine, and sulfadoxine-pyrimethamine have been reported, mostly in endemic regions, including Indonesia [5]. A study in 2011 showed that in 1973–2005 and 1974–2010, 52% of *Plasmodium falciparum* and 48% of *P. vivax* positive samples were resistant to chloroquine. Chloroquine, a weak base, is protonated once it enters the digestive vacuole (DV) of parasites with acidic pH. This protonated chloroquine binds to heme that is toxic to the parasite, which is a byproduct of hemoglobin digestion inside DV, and is normally converted into nontoxic hemozoin by the parasite. The primary driver of chloroquine resistance is a mutation in the *P. falciparum chloroquine resistance transporter* gene (*pfCRT*) and the *P. vivax chloroquine resistance transporter* gene (*pvCRT*), which enables the efflux of protonated drug molecules from DV; thus, it will not bind to its target, heme. Another factor that contributed to chloroquine resistance is a mutation in the *P. falciparum multidrug resistance* (*pfMDR1*) gene and its ortholog *P. vivax multidrug resistance* (*pvMDR1*) gene in *P. vivax*, which reduced the transport of multidrugs, including chloroquine, into DV and leads to drug resistance [6,7].

In vivo and in vitro studies of *Plasmodium falciparum* from 1985 to 2011 have shown that 22% and 63%, respectively, were resistant to sulfadioxine-pyrimethamine and at least 6% were resistant to quinine in vitro [8]. The resistance to quinine is linked to amplified *pfMDR1*, whereas sulfadioxine-pyrimethamine resistance is linked to the *P. falciparum dihydrofolate reductase* (*pfDHFR*) and *P. falciparum dihydroprotease (pfDHPS*) gene mutations, which are the sites of action for sulfadioxine and pyrimethamine, respectively [6,9]. The latest report from the WHO regarding artemisinin resistance showed partial artemisinin resistance, which was defined as delayed parasite clearance detected in SEA, particularly in the Greater Mekong subregion, and in Africa, particularly in Rwanda and Uganda [1]. Artemisinin and its derivates affect a multitude of organellar and cellular processes including hemoglobin endocytosis, glycolysis, protein synthesis and degradation, and cell cycle regulation. The cleavage of its endoperoxide bridge by Fe-phytoporphyrin IX (Fe^2+^-heme) from parasite-digested hemoglobin activates artemisinin. The Fe^2+^-heme-artemisinin radicals then alkylate heme, protein, and lipids to amplify cytotoxic-reactive oxygen species, ultimately leading to cell death. The resistance of artemisinin is linked to the mutation of the parasite’s *k13* gene (*kelch13*). Lowered *k13* levels lead to a reduction in hemoglobin endocytosis, therefore, the digestion is reduced and a low level of Fe^2+^-heme presence leads to lesser activation of artemisinin [6]. These antiplasmodial agent resistance occurrences suggest that the discovery of a potential new drug with another mechanism might play an important role in overcoming emerging drug resistance.

Natural products have played an important role in drug development over the centuries, including in the development of antimalarial drugs. Quinine, a quinoline alkaloid from *Cinchona* sp., and artemisinin, a sesquiterpene lactone from *Artemisia* sp., are the best examples of antimalarial drugs developed from natural products. A study reported that >60% of antiparasitic new chemical entities identified from 1981 to 2014 were related to natural products [10]. Indonesia, having the second highest number of indigenous medicinal plants after the Amazon rainforest, has huge potential as a source of new antiparasitic drugs; however, Indonesia has not yet taken full advantage of this potential [11,12]. Indonesia is particularly unique in terms of medicinal plant utilization because the archipelago consists of 17,000 islands and approximately 6000 are inhabited, which leads to indigenous knowledge of traditional medicine and medicinal plants between the islands and ethnicities, including Papua Island where malaria is still high [4,13].

Recent research on traditional medicine used as antimalarial treatments on Papua Island, Indonesia, showed that 72 plant species have been used as traditional medicine. Among them, Pulai (*Alstonia scholaris* (L.) R. Br.), Papaya (*Carica papaya* L.), Sambiloto (*Andrographis paniculata* (Burm. f.) Nees), and Ciplukan (*Physalis minima* L.) were the most frequently mentioned by the locals to treat malaria [14]. Thus, this review highlights the current research status of these plants for potential novel antiplasmodial development.

## 2. Methods

**Figure 1:**
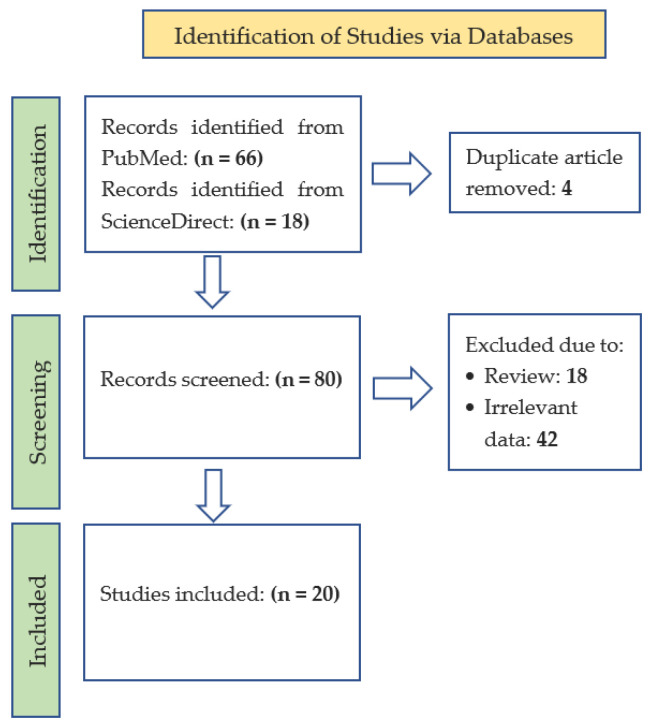
Flow diagram of the study.

Literature searches were performed in PubMed and ScienceDirect. The following search keywords were used in PubMed for each plant: (antimalarial[Title/Abstract] OR antiplasmodial[Title/Abstract]) AND (alstonia scholaris[Title/Abstract]); (antimalarial[Title/Abstract] OR antiplasmodial[Title/Abstract]) AND (carica papaya[Title/Abstract]); (antimalarial[Title/Abstract] OR antiplasmodial[Title/Abstract]) AND (andrographis paniculata[Title/Abstract]); and (antimalarial[Title/Abstract] OR antiplasmodial[Title/Abstract]) AND (physalis minima[Title/Abstract] OR physalins[Title/Abstract]). Keywords used in ScienceDirect included Alstonia scholaris AND (antimalarial OR antiplasmodial), Carica papaya AND (antimalarial OR antiplasmodial), andrographis paniculata AND (antimalarial OR antiplasmodial), and Physalis minima AND (antimalarial OR antiplasmodial), all in title, abstract, and keywords. The flow diagram is shown in Figure 1.

## 3. *Alstonia scholaris* (L.) R. Br.

**Figure 2:**
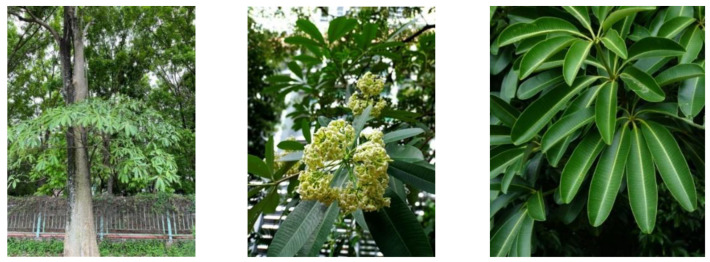
*Alstonia scholaris* (L.) R.Br. [17,18,19].

*A. scholaris* of the Apocynaceae family is a large, buttressed, evergreen tree that is 6–10 m in height (Figure 2). It is widespread in the tropical parts of Asia and Africa [15,16]. It is commonly known as the Devil’s tree, milkwood pine, and white cheese wood [15]. In Indonesia, it is known as pulai and locally in Papua Island, it is known as arasue, pohon susu, kayu susu, igeii, aikahahe, ibuong, and yepaa [14].

### 3.1. Ethnopharmacology

In various traditional medicines, *A. scholaris* has been used as and is considered an important medicinal plant. It is commonly used to treat fever, diarrhea, malaria, dysentery, dyspepsia, asthma, bronchitis, as an antidiabetic, etc. [20,21]. In the Indian traditional medicine system, Ayurveda, the bark is used as a tonic, alterative, febrifuge, and gastrointestinal sedative. It is also useful in the treatment of malarial fever [22]. In traditional Chinese medicine, the leaf is used to treat chronic respiratory diseases [23]. In Indonesia, mainly on Papua Island, the bark is used to treat fever and malaria. According to the Papua Health Department, local people use dried bark. It was soaked overnight and one glass is consumed daily to combat malaria, until the disease is cured. Alternatively, fresh bark is chewed directly, and then the liquid or juice is swallowed. However, how much bark should be used as medication has not been mentioned [14,24].

### 3.2. Phytochemistry

*A. scholaris* is rich in alkaloids, mainly indole alkaloids. The bark contains several substances, such as ditamine, echitamine, echitenine, echicaoutchin, echicerin, echitin, echitein, echiretin, ditain, ditamine, losbanine, 6,7-secoangustilobine B, N^b^-demethyl echitmaine, 17-*O*-acetyl echitamine, picraline deacetyl, lupeol, β-sitosterol, alstonidine, alstonine, villalstonine, and macrocarpamine [22,25]. The leaf contains alkaloid compounds, such as scholaricine, 19-epischolaricine, vallesamine, picrinine, (19,20) *E*-alstoscholarine, (19,20) *Z*-alstoscholarine, 5-methoxyaspidophylline, picralinal, 5-methoxystrictamine, scholarisine A, scholarisines H-O, (±)-scholarisine II, alstorisine A, scholarisines B-G, normavacurine-21-one, 5-hydroxy-19, 20-*E*-alschomine, 5-hydroxy-19, 20-*Z*-alschomine, altoscholarisines A-J, alstoniascholarines A-K, alstoniascholarines L-Q, alstolactines A-C, and alstonitrine A [16,26,27,28,29,30,31,32,33,34,35,36,37,38,39,40]. The leaf also contains non-alkaloids, namely, scholareins A-D, megastigmane-3β, 4α, 9-triol, and 7-megastigmene-3,6,9-triol [41,42]. *A. scholaris* compounds are well-investigated thus it could be advantageous to explore known compounds for their pharmacological activities.

### 3.3. Antiplasmodial Activity

**Figure 3:**
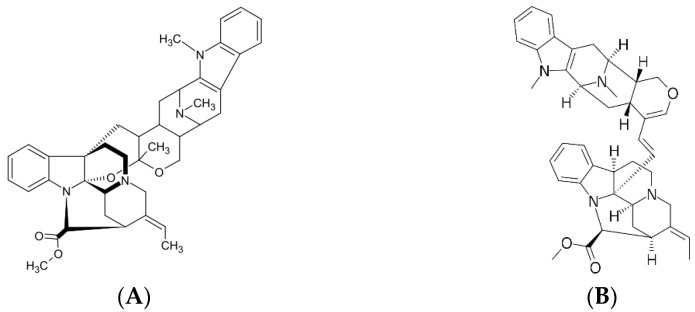
Structures of villalstonine (**A**) and macrocarpamine (**B**).

Several in vitro and in vivo studies have investigated the antiplasmodial activities of *A. scholaris*. Two in vitro studies on the chloroquine-sensitive *P. falciparum* strain 3D7 were performed by Taek et al. (2021) and Abdillah et al. (2015) who investigated the activity of stem bark extract. The only difference in their method was the solvent used in the extraction by maceration, which were 95% and 70% ethanol. However, a quite significant difference was found in the IC_50_ result, where extraction with 70% ethanol gives a strong antiplasmodial activity (0.165 µg/mL) compared to 95% ethanol with only moderate activity (15.46 µg/mL) [43,44]. In 2006, Ouattara et al. categorized an extract as very active or strong antiplasmodial if the IC_50_ is <5 μg/mL, active or moderate antiplasmodial if IC_50_: 5–50 μg/mL, less active or weak antiplasmodial if IC_50_: 50–100 μg/mL, and inactive if IC_50_: >100 μg/mL [45]. However, what caused the difference is still unclear. Only Abdillah et al. conducted phytochemical screening; thus, no comparison can be performed. However, the difference in the activity of 70% and 96% ethanol extract showed that more polar compounds that should have been extracted better by 70% ethanol might have demonstrated better in vitro antiplasmodial activity. The environment where the plant grows also plays an important role, as it could result in different chemical compositions in plants. Keawpradub et al. (1999) compared the in vitro antiplasmodial activity against the *P. falciparum* multidrug-resistant K1 strain from a methanolic extract using leaves, stem bark, and root bark of three *Alstonia* species from Thailand. The result showed that the stem bark of *A. scholaris* was second best with the lowest IC_50_ of 181.4 µg/mL, which is considered inactive, much weaker than the root bark of *Alstonia macrophylla* with the lowest IC_50_ of 5.7 µg/mL [45,46]. This result suggested that the methanolic extract of *A. scholaris* might not well deal with the *P. falciparum* multidrug-resistant strain, although other studies on the chloroquine-sensitive strain gave a strong and moderate activity [43,44]. This study also tested villalstonine and macrocarpamine which were isolated from *A. macrophylla* and well known to be present in *A. scholaris*. Interestingly, these isolates gave a strong activity with IC_50_ of 0.27 and 0.36 µg/mL, respectively. This could indicate that *A. macrophylla* might contain higher levels of villalstonine and macrocarpamine (Figure 3) than *A. scholaris*, although further research is needed for confirmation. The author suggested that these active alkaloids might act through a different mechanism to chloroquine and further investigation is needed to provide evidence [46]. The possible mechanism of bisindole alkaloids is the inhibition of hemozoin formation but in a manner different from chloroquine; hence, this explains the activity against chloroquine-resistant *P. falciparum* [47].

The in vivo activity of *A. scholaris* stem bark has also been studied. Abdullah et al. (2015) continued their previous in vitro study against the *Plasmodium bergheii* NK 65 strain in mice. The ethanolic extract of *A. scholaris* stem bark gave an ED_50_ of 121.94 mg/kg which was categorized as good activity [44]. According to Muñoz et al. (2000), in vivo antiplasmodial activity is classified as excellent if ED_50_ is ≤100 mg/kg BW/day, good if ED_50_ is ≤101–250 mg/kg BW/day, moderate if ED_50_ is 251–500 mg/kg BW/day, and inactive if ED_50_ is >500 mg/kg BW/day [48]. In addition, in an in vivo study, Intan et al. investigate the activity of a mixed extract of *A. scholaris* and *Phyllanthus niruri* (1:1). The extract was obtained by maceration with 70% ethanol, which reduced parasitemia and differential leukocyte count at a dose of 147.78 mg/kg [49]. Both studies have shown that the 70% ethanol extract of *A. scholaris* exerted a good in vivo antiplasmodial effect, although the second study used a mixture of two plant extracts. From the studies discussed, stem bark ethanolic extract should be the focus of further investigation against the *P. falciparum* chloroquine-resistant strain and the isolate, villalstonine, and macrocarpamine should be subjected to further in vivo study. The in vitro and in vivo studies of *A. scholaris* are summarized in Table 1.

The toxicity profile of *A. scholaris* has been studied for its methanolic and hydroalcoholic extract. Bello et al. (2016) conducted the acute and subacute toxicity studies of methanolic extract in Sprague–Dawley rats. In the acute study, the extract, at a dose of 2000 mg/kg BW, showed no toxicity, whereas in the subacute study, two female rats were recorded to have died at the highest dose of 1000 mg/kg BW after 28 days of oral administration. In histopathological studies, liver damage was found at the long-term administration of a high dose [50]. Baliga et al. (2004) investigated the acute and subacute toxicity of the hydroalcoholic extract of *A. scholaris* in mice and rats. In an acute study, the oral administration of 2000 mg/kg BW to Swiss albino mice showed no toxicity, whereas 1100 mg/Kg BW administered intraperitoneally resulted in animal deaths. The subacute toxicity test showed 30% mortality at a dose of 240 mg/kg BW given intraperitoneally, with damage in all organs [51]. From this study, oral administration of *A. scholaris* methanolic and hydroalcoholic extracts, as the administration route of the activity studies, showed low toxicity according to the OECD 425 guidelines [52].

**Table 1:** Summary of Antiplasmodial Activity of *A. scholaris*, *C. papaya*, *A. paniculata*, and *P. minima*.

Plant	Plant’s Part Used	Solvent	Extraction Method	Assay	Dose/ED_50_/IC_50_	Active Compound	Reference
*Alstonia scholaris*	Stem Bark	Ethanol 95%	Maceration (triplicate)	In vitro (*P. falciparum* chloroquine-sensitive 3D7 strain)	IC_50_: 15.46 µg/mL (Moderate)	-	[43]
	Stem Bark	Ethanol 70%	Maceration (triplicate)	In vitro (*P. falciparum* chloroquine-sensitive 3D7 strain)	IC_50_: 0.165 µg/mL (Strong)	-	[44]
				In vivo (*P. berghei*, NK 65 strain in mice)	ED_50_: 121.94 mg/kg BW (Good)	-	
	Leaves	Methanol	Percolation	In vitro (*P. falciparum* chloroquine-resistant K1)	IC_50_: 210.8 µg/mL	-	[46]
	Stem Bark				IC_50_: 181.4 µg/mL	-	
	Root Bark				IC_50_: 370.2 µg/mL	-	
	Isolates *	-	-		IC_50_: 0.27 µg/mL (1) IC_50_: 0.36 µg/mL (2)	(1) Villalstonine (2) Macrocarpamine	
*Carica papaya*	Leaves	Ethanol 70%	Maceration (triplicate)	In vitro (*P. falciparum* chloroquine-sensitive 3D7 strain)	IC_50_: 0.177 µg/mL (Strong)	-	[44]
				In vivo (*P. berghei*, NK65 strain in mice)	ED_50_: 173.20 mg/kg BW (Good)	-	
	Leaves	Ethanol	Percolation	In vitro (*P. falciparum*)	IC_50_: 46.23 µg/mL	-	[53]
	Stem				IC_50_: 65.13 µg/mL	-	
	Leaves	Methanol 50%	Not mentioned	In vivo (*P. berghei*, NK65 strain in mice)	Dose: 100 mg/kg BW (>50% chemosuppresion)	-	[54]
	Leaves	Sequential solvent: petroleum ether followed by dichlorometane, ethyl acetate, methanol, and water	Maceration (4-5 times, shaken continuously)	In vitro (*P. falciparum* chloroquine-sensitive D10)	IC_50_: 2.6 µg/mL (ethyl acetate) IC_50_: 10.8 µg/mL (methanol) IC_50_: 12.8 µg/mL (dichloromethane) IC_50_: 16.4 µg/mL (petroleum ether) IC_50_ > 50 µg/mL (water)	-	[55]
	Isolates	-	-	In vitro (*P. falciparum* chloroquine-sensitive D10)	IC_50_: 3.58 µg/mL (1) IC_50_: 6.88 µg/mL (2)	(1) Linolenic Acid (2) Linoleic Acid	
				In vitro (*P. falciparum* chloroquine-resistant Dd2 strain)	IC_50_: 4.40 µg/mL (1) IC_50_: 6.80 µg/mL (2)		
	Isolate	-	-	In vitro *(P. falciparum)*	IC_50_: 0.2 µM	Carpaine	[56]
				In vivo (*P. berghei* ANKA strain)	Dose: not specified, resulting only 11.9% reduction in parasitaemia	Carpaine	
	Isolate	-	-	In vitro (*P. falciparum* chloroquine-sensitive 3D7)	IC_50_: 2.01 µg/mL	Carpaine	[57]
				In vitro (*P. falciparum* chloroquine-resistant Dd2 strain)	IC_50_: 2.19 µg/mL		
	Fruit rind/peel	Chloroform	Soxhlet	In vivo (*P. berghei* chloroquine-sensitive ANKA strain)	Dose: 400 mg/kg BW (61.78% Chemosuppresion, moderate)	-	[58]
		Methanol			Dose: 400 mg/kg BW (37.65% Chemosuppresion, Mild)	-	
		Petroleum ether			Dose: 400 mg/kg BW (18.39% Chemosuppresion, Weak)	-	
	Root	Chloroform	Soxhlet	In vivo (*P. berghei* chloroquine-sensitive ANKA strain)	Dose: 400 mg/kg BW (43.77% Chemosuppresion, Mild)	-	
		Methanol			Dose: 400 mg/kg BW (48.11% Chemosuppresion, Mild)	-	
		Water			Dose: 400 mg/kg BW (25.63% Chemosuppresion, Mild)	-	
	Leaves	Water	Maceration	In vivo (*P. berghei*, NK65 strain in mice)	Dose: 350 mg/kg BW	-	[59]
	Leaves	Ethanol	Soxhlet	In vitro (*P. falciparum* chloroquine-sensitive)	IC_50_: 25–150 µg/mL	-	[60]
				In vitro (*P. falciparum* chloroquine-resistant)	IC_50_: 25–150 µg/mL	-	
*Andrographis paniculata*	Herb (aerial parts)	Purified ethyl acetate fraction from Ethanol 96% extract	Maceration followed by liquid-liquid fractionation and further purification	In vivo (*P. berghei* chloroquine-sensitive ANKA strain)	Dose: 15 mg purified fraction per 300 mg tablet by wet granulation (78.16% inhibition) and 60 mg purified fraction per 150 mg tablet by solid dispersion method (80.35% inhibition)	-	[61]
	Herb (aerial parts)	Ethyl acetate fraction from ethanol 96% extract	Maceration followed by liquid-liquid fractionation	Clinical trial Phase 1	Equivalent to 35 mg andrographolide per 380 mg granule. 4 tablets twice daily for 4 days, classified as non-toxic & safe	-	[62]
	Herb (aerial parts)	Ethanol 50%	Percolation	In vitro (*P. falciparum* of Papua strain (2300)	Optimal dose at 200 µg/mL (10% parasitemia)	-	[63]
	Not mentioned	Ethanol 50%	Percolation	Clinical trial Phase 2	Dose: 250 mg extract per 460 mg capsule → 94.2% (65 of 69) patients have negative parasitaemia on day 7	-	[64]
	Herb (aerial parts)	Methanol	Stirred at 4 °C overnight	In vitro (*P. falciparum* chloroquine-sensitive MRC-pf-20 and chloroquine-resistant MRC-pf-303)	IC_50_: 7.2 µg/mL for both strain	-	[65]
				In vivo (*P. berghei* ANKA strain)	Dose: 7 mg/Kg BW, 39% parasitaemia at 12th day, control mice all died		
	Isolate	-	-	In vitro (*P. falciparum* chloroquine-resistant MRC-pf-303)	IC_50_: 9.1 µM	Andrographolide	[66]
				In vivo (*P. bergheii* ANKA strain)	Dose: 15 mg/Kg BW, 46% parasitaemia at day 13–15, control mice all died		
	Isolate	-	-	In vitro (*P. falciparum* 3D7)	IC_50_: 13.70 µM	Andrographolide	[67]
				In vivo (*P. berghei* chloroquine-sensitive NK65)	Dose: 5 mg/kg BW, 60.17% chemosuppresion		
	Whole plant	*n*-Hexane	Soxhlet	In vitro (*P. falciparum* FCR-3 strain)	-	-	[68]
		Chloroform			100% inhibition at 0.05 mg/mL after 24 h		
		Methanol			100% inhibition at 25 mg/mL after 48 h		
	Whole plant	*n*-Hexane	Soxhlet	In vivo (*P. berghei* ANKA)	-	-	
		Chloroform			-		
		Methanol			5 mg/Kg BW → delayed parasetaemia and all died at day 7 where control died at day 5		
*Physalis minima*	Isolate *	-	-	In vitro (*P. falciparum* W2 clone)	IC_50_: 2.8 µg/mL	Physalin B	[69]
					IC_50_: 55 µg/mL	Physalin D	
					IC_50_: 2.2 µg/mL	Physalin F	
					IC_50_: 6.7 µg/mL	Physalin G	
				In vivo (*P. berghei* strain NK65)	Dose: 100 mg/kg → no decrease in parasitemia	Physalin F	
					Dose: 100 mg/kg → 65% decrease in parasitemia	Physalin D	

* Isolate(s) were obtained from other plant species, but also present in the discussed plants.

## 4. *Carica papaya* L.

**Figure 4:**
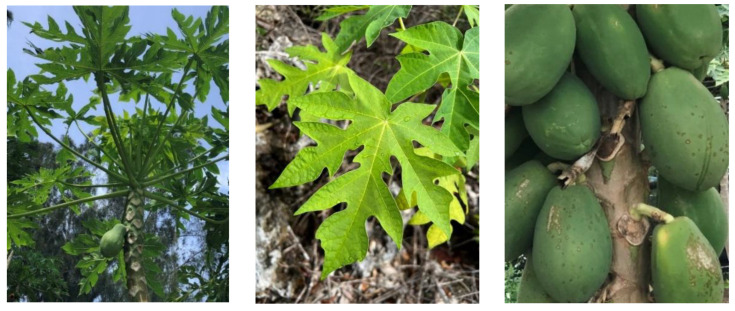
*Carica papaya* L. [71,72,73].

*C. papaya* of the Caricaceae family is a tree or shrub 8–10 m in height (Figure 4). It is native to tropical America but is cultivated all over the tropics and subtropics [70]. It is traditionally widely used as a treatment. In Indonesia, it is generally known as Pepaya. In Papua Island it is also locally known as peceren, eteradado, monofo, papaya, labseren, ogoseren, cacaveka, mbikin, senene ranau, and pepaya bllah [14].

### 4.1. Ethnopharmacology

*C. papaya* is used as a treatment for various conditions. Its leaf is used to treat dengue, inhibit cancer growth, treat malaria, for digestion problems and acne, increased appetite, to ease menstrual pain, relieve nausea, jaundice, kill viruses, and modulate immune response [14,74]. The fruit is used as a laxative and a treatment for indigestion [74]. The seed is used as a contraceptive for men and for abortion in women [75]. The flowers are utilized as digestive disorder treatments, diuretics, and tonics. In Papua Island, *C. papaya* leaf is used to combat malaria. In Waibon village, locals squeeze the yellowish leaves, add a pinch of salt, and consume the mixture orally. In other villages, young leaves are boiled with three glasses of water until reduced to one glass and then consumed orally [14,76].

### 4.2. Phytochemistry

Various parts of *C. papaya* are reported to contain caffeic acid, myricetin, quercetin, papain, α-tocopherol, benzyl isothiocyanate, kaempferol, squalene, phytol, campesterol, stigmasterol, β-sitosterol, hexadecanoid acid, γ-tocopherol, olean-12-ene, 13,17-cyclocursan-3-one, cycloartenol, and carpaine [77,78]. Another study characterized *C. papaya* seed oil and reported that it contains oleic, palmitic, stearic, linoleic, palmitoleic, arachidic, gadoleic, myristic, and margoric acid [79].

### 4.3. Antiplasmodial Activity

Antiplasmodial studies on different parts of *C. papaya* have been performed. Three studies used different extraction methods and examined the in vitro antiplasmodial activity of leaves. Abdillah et al. (2015), Ravikumar et al. (2012), and Kovendan et al. (2012) used maceration, percolation, and soxhlet extraction methods, respectively, with ethanol as solvent, and all were tested against *P. falciparum*. The IC_50_ of the ethanolic extract by maceration, percolation, and soxhlet methods were 0.18, 46.23, and 25 µg/mL, respectively [44,53,60]. Results varied, as the activity of the ethanolic extract obtained with the maceration method was classified as strong, while those with percolation and Soxhlet methods were classified as moderate [45]. A noticeable difference was due to the *P. falciparum* strain used. Abdillah et al. used the chloroquine-sensitive strain, Kovendan et al. used chloroquine-sensitive and resistant strains, whereas Ravikumar et al. did not mention the strain used. Another distinctive factor might be the concentration of ethanol used. Abdillah et al. used 70% ethanol, whereas the other studies did not mention the concentration. The difference in the concentration might well give a different chemical composition. The studies by Abdillah et al. and Kovendan et al. were compared since both performed phytochemical screening. Alkaloids, flavonoids, saponins, tannins, and triterpenoids were detected in both extracts. Abdillah et al. found coumarins, quinones, and steroids, but Kovendan et al. did not find these compounds. This might have resulted in the difference in activities, although more qualitative and quantitative compound analyses are needed.

**Figure 5:**
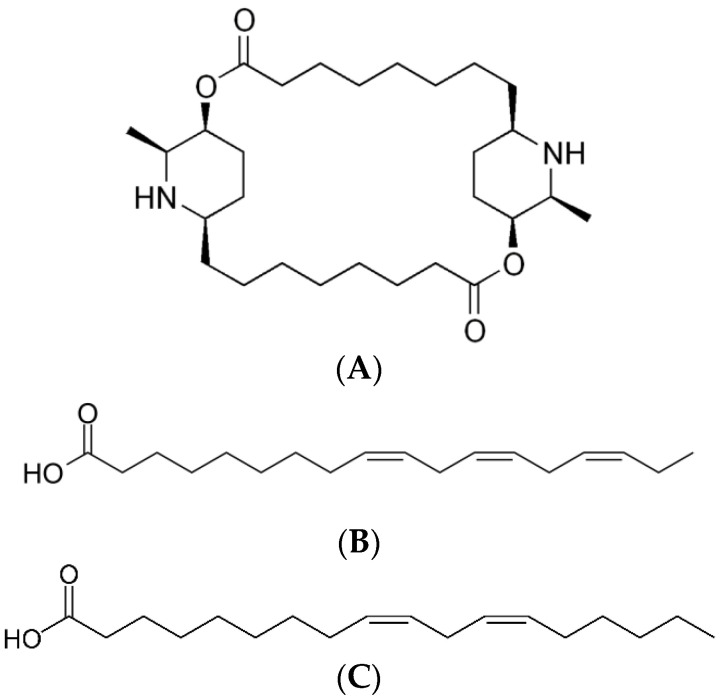
Structures of carpaine (**A**), linolenic acid (**B**), and linoleic acid (**C**).

Moreover, the stem was also subjected to in vitro testing by Ravikumar et al. using percolation extraction with ethanol extract; however, it demonstrated less activity with an IC_50_ of 65.13 µg/mL [53]. In addition, the in vitro activity in *C. papaya* leaves was investigated by Teng et al. (2019), who reported that the alkaloidal *n*-hexane extract was the most active against *P. falciparum* 3D7 and Dd2 strains with IC_50_ of 3.45 and 1.52 µg/mL, followed by dichloromethane extract with IC_50_ of 7.67 and 4.50 µg/mL, respectively. Carpaine (Figure 5A) was isolated from alkaloidal *n*-hexane extract and further screened against the *P. falciparum* 3D7 strain. It showed better activity with an IC_50_ of 2.01 µg/mL, suggesting the bioactive compound from the leaves [57]. Julianti et al. (2014) also isolated and tested carpaine in vitro against *P. falciparum*, which showed strong activity with an IC_50_ of 0.2 µM [56]. Melariri et al. (2011) tested several extracts obtained by sequential extraction and showed that the ethyl acetate extract was the most active with 2.6 µg/mL against *P. falciparum* chloroquine-sensitive D10 strain. Two isolates, linolenic acid and linoleic acid (Figure 5B,C) were then obtained and tested, which gave strong and good activity against *P. falciparum* chloroquine-resistant with IC_50_ of 3.58 and 6.88 µg/mL, respectively [55]. A previous study reported that fatty acids could enhance the neutrophil-mediated killing of the asexual blood forms of *P. falciparum* and that they might be a possible mechanism for linolenic and linonelic acid activity, although further confirmation is needed [80].

In vivo activities in three different plant organs were investigated by four research groups. In vivo assays for leaf extracts were investigated by Abdillah et al. (2015), Atanu et al. (2021), and Okpe et al. (2016). All studies used the maceration method, but with different solvents, namely 70% ethanol, 50% methanol, and water, respectively [44,54,59]. Abdillah et al. reported an ED_50_ of 173.2 mg/kg BW. According to the classification by Munoz et al., the activity of *C. papaya* ethanolic leaf extract was classified as good [48]. Atanu et al. and Okpe et al. suggest that the extracts gave suppressed parasitism by >50% at doses of 100 and 350 mg/kg BW, respectively [54,59]. Munoz et al. also stated that in vivo antiplasmodial activity can be classified as moderate, good, and very good if an extract suppressed parasitemia by >50% at doses of 500, 250, and 100 mg/kg per BW/day, respectively [48]. Based on that classification, the activities of methanolic and aqueous extract were considered very good and moderate, respectively. However, Okpe et al. only tested for one dose at a time, and the parasite suppression result was on par with the positive control (halofantrine 25 mg/kg BW); therefore, it is still possible to achieve parasite suppression ≥50% with a lower dose [59]. In addition, Julianti et al. (2014) investigated the carpaine isolate for its in vivo antiplasmodial activity; however, the results were somewhat disappointing as the treatment only reduced parasitemia by 11.9% despite the promising result of an in vitro study [56]. However, the exact dose was not mentioned; thus, we cannot conclude whether the low activity was due to the low dose given or other factors. From these studies, *C. papaya* leaf extract showed good potential as an antiplasmodial agent; however, the active compound must be further studied given the contrasting results of Carpaine in vitro and in vivo. In addition, the possibility of a synergistic effect of the compounds contained in the extract should also be considered. Other candidates as bioactive compounds should be considered and further examined because strong in vitro activity against chloroquine-resistant *P. falciparum* was found.

The fruit rind and roots of *C. papaya* were also studied for their in vivo antiplasmodial activities. In this study, Zeleke et al. (2017) obtained extracts through the Soxhlet method using three solvents, namely petroleum ether, chloroform, and methanol. The doses used were 400 mg/Kg BW. Petroleum ether, chloroform, and methanol extracts of fruit rind showed parasite suppression rates of 61.78%, 37.65%, and 18.39%, and extracts from roots had rates of 43.77%, 48.11%, and 25.63%, respectively. Only petroleum ether extract achieved a parasite suppression rate of >50%, which was classified as good [48,58]. Based on all these studies, the fruit rind and roots of *C. papaya* likely have less potential as antiplasmodial agents compared with leaves as summarized in Table 1. The antiplasmodial mechanism is still unclear; however, macrocylic dilactone alkaloids such as carpaine might work as a cation-chelating agent based on their structure [81]. From these studies, a direct comparison between methanolic, ethanolic, and ethyl acetate extracts might be performed against the *P. falciparum* chloroquine-resistant strain as the result from studies available varies. Moreover, the constituents of the most active extract should be determined to investigate the possibility of its synergistic action. Further in vivo studies must confirm the in vitro activity of the extracts and linolenic acid, which posed a strong activity in vitro against both *P. falciparum* chloroquine-sensitive and resistant strains.

The animal toxicity study of *C. papaya* leaf was summarized by Lim et al. (2021) in a systematic scoping review where, generally, leaf juice and aqueous extracts at doses up to 2000 mg/Kg are nontoxic in rats in acute and subacute studies [82,83,84]. The hydroalcoholic extract also showed very low toxicity with an LD_50_ of 5120 mg/kg BW when administered orally in chicks and no toxicity was noted in oral subchronic administration at a low dose [85]. The methanol sub-fraction from the chloroform extract of seeds showed no developmental toxicity or teratogenicity at the highest dose of 500 mg/kg BW [86]. The clinical evidence also suggests that oral consumption of *C. papaya* leaf, which is mostly aqueous extract or juice, is well tolerated [84].

## 5. *Andrographis paniculata* (Burm. f.)

**Figure 6:**
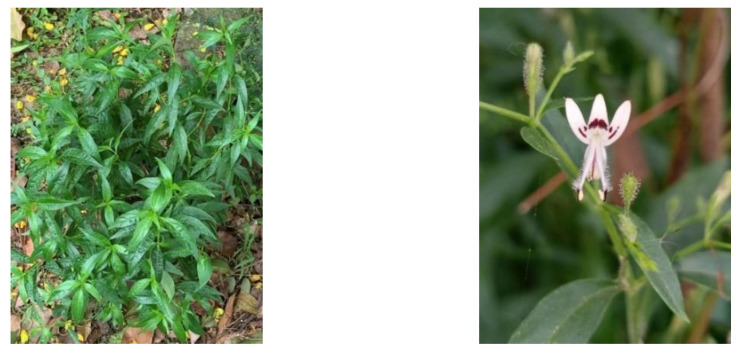
*Andrographis paniculata* (Burm. f) [92,93].

*A. paniculata* of the Acanthaceae family is an herb that is up to 50 cm tall, annual, branched, and extremely bitter in all parts of the plant body (Figure 6) [87,88]. It is native to Taiwan, mainland China, India and Sri Lanka, is widely found in southern and southeast Asia, such as Bangladesh, China, Indonesia, Malaysia, Myanmar, Philippines, and Thailand [88,89,90,91]. It is commonly known as the king of bitter or kalmegh and, in Indonesia, it is known as sambiloto, sambiroto, and samiroto [14].

### 5.1. Ethnopharmacology

Commonly, aerial parts of *A. paniculata* are used to treat various infectious diseases such as leprosy, gonorrhea, respiratory tract infections, scabies, malaria, helminthiasis, herpes, peptic ulcers, and is used topically for skin infection and snake bites. It is also used as a treatment for irregular bowel habits, loss of appetite, alopecia, diabetes, jaundice, dyspepsia, and coughs [88,89,90,91,94]. In China, it was described as bitter and cold and considered antipyretic, detoxicant, anti-inflammatory, and detumescent. It is also thought to be used to remove “pathogenic heat” from the blood [90]. In Papua, *A. paniculata* is used to combat malaria. They use 10–15 leaves of sambiloto, boil them with water, and drink them two times a day until the disease is cured [24].

### 5.2. Phytochemistry

The main compound of *A. paniculata* is andrographolide, a diterpenoid lactone. Other known diterpenoid compounds are neoandrographolide, isoandrographolide, bis-andrographolides A-D, 14-deoxy-11-oxoandrographolide, 14-deoxy-11,12-didehydroandrographolide, 14-deoxyandrographolide, andrograpanin, andrographiside, andrographane, andrographic acid, andrographidines A-F, andrographidoids A-E, andropaniosides A & B, andrographatoside, andrographolactone, andopaniculosin A, andropaniculoside A, andropanolide, dehydroandrographolide, and paniculides A-C [95,96,97,98,99,100,101,102,103,104].

Some flavonoids are also found in *A. paniculata*, such as apigenin, cosmosiin, isoswertisin, luteolin, quercetin, skullcapflavone I, and onysilin [105]. Other constituents found in *A. paniculata* include α & β-sitosterol, β-daucosterol, caffeoylquinic acid, caffeic acid, cinnamic acid, ferulic acid, feruloylquinic acid, oleanolic acid, quinic acid, stigmasterol, and trans-cinnamic acid [96,106,107,108].

### 5.3. Antiplasmodial Activity

Among these four plants, *A. paniculata* is the most well studied for its antiplasmodial activity. In vitro studies were conducted by three research groups. Rahman et al. (1999) performed in vitro testing of the whole *A. paniculata* plant sequentially extracted by the Soxhlet method with *n*-hexane, chloroform, and methanol. Only chloroform and methanol extracts were tested and the results showed that the chloroform extract at a dose of 0.05 mg/mL had an inhibition rate of 100% after 24 h and at a dose of 0.025 mg/mL showed 100% inhibition after 48 h. The methanol extract at a dose of 0.4 mg/mL showed 100% inhibition after 24 h and at 0.025 mg/mL showed 100% inhibition after 72 h. However, the IC_50_ value was not determined [68]. Mishra et al. (2009) investigated the in vitro antiplasmodial activity of aerial parts of *A. paniculata*, extracted by the maceration method at 4 °C overnight with 98% methanol and tested against two strains of *P. falciparum*, the chloroquine-sensitive (MRC-pf-20) and choloroquine-resistant (MRC-pf-303). The IC_50_ was 7.2 µg/mL which, according to Ouattara et al., was considered strong or very active [45,65]. The results showed that the extract gave good activity for both strains, suggesting that the extract could well deal with the chloroquine-resistance mechanism of *P. falciparum* [65]. In another study, Zein et al. (2013) investigated the in vitro activity of *A. paniculata* extract through the maceration method for 3 h with 50% ethanol as a solvent against *P. falciparum* of Papua strain (2300). Accordingly, at a dose of 200 µg/mL, the parasite density was 10% compared with 38.7% in the control. However, this study also did not calculate the IC_50_ value [63].

**Figure 7:**
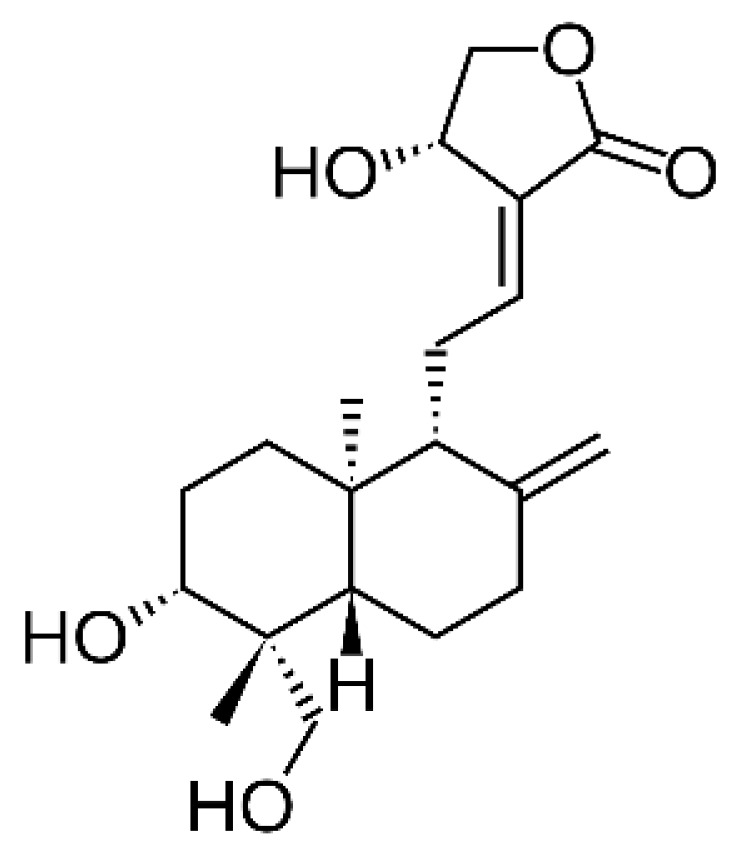
Structure of andrographolide.

The investigation of the in vivo activity was performed by Rahman et al. (1999), which further tested the methanolic extract for its in vivo antiplasmodial activity against *P. berghei*. At a dose of 5 mg/kg administered intraperitoneally for 4 days, the extract group showed delayed parasitemia where all mice died on day 7, compared with the control group without treatment where all mice died on day 5 [68]. This result is different to that reported by Mishra et al. (2009), who continued their investigation in vitro to evaluate the in vivo activity of the methanolic extract. Accordingly, at a dose of 7 mg/Kg BW, 39% parasitemia was observed on day 12, suggesting good suppression [65]. These two studies differed in the extraction method used. Sequential extraction by the Soxhlet method with *n*-hexane, chloroform, and methanol appeared to reduce the bioactive content in methanolic extract in the study by Rahman et al. (1999), compared with direct soaking overnight at 4 °C by Mishra et al. (2009), which appeared to be a better extraction method. In addition, the oral administration route appears to be superior to intraperitoneal administration [65,68]. Another in vivo study was performed by Widyawaruyanti et al. (2014), who used a purified ethyl acetate fraction from a 96% ethanol extract in tablet form. The study showed that 15 mg pure fraction per 300 mg tablet by wet granulation resulted in 78.16% parasite suppression and 60 mg pure fraction per 150 mg tablet by the solid dispersion method achieved 80.35% parasite suppression which, according to Munoz et al., was classified as very good [48,61]. The same research group further conducted a double-blind controlled crossover clinical trial (phase 1) for this pure fraction in 30 healthy volunteers, with a dose equivalent to 35 mg andrographolide per tablet. The study reported no difference in vital signs, hematological, and biochemical parameters when compared with the control group (placebo). In addition, increased appetite and better sleep were reported after the administration of the test tablet, which could be beneficial in uncomplicated malaria in which one of the symptoms is anorexia [62,109]. Hassan et al. (2019) studied the isolate, investigating the in vitro and in vivo activities of andrographolide (Figure 7). The IC_50_ of andrographolide against the *P. falciparum* 3D7 strain was 13.70 µM or roughly 4.8 µg/mL when converted, as the molar mass of andrographolide is 350.45 g/mol which was considered strong. The in vivo activity against *P. berghei* in infected mice with a dose of 5 mg/kg BW achieved 60.17% parasite suppression and, according to Munoz et al., it is considered very good [45,48,67]. This study is in line with the previous study by Mishra et al. (2011), in which the in vitro activity against the *P. falciparum* chloroquine-resistant strain was considered strong with an IC_50_ of 9.1 µM. The in vivo study against *P. berghei* at a dose of 15 mg/kg showed parasitemia level at 46% (>50% suppression) on day 13, where all controls died [66]. This shows that andrographolide could be the bioactive compound of *A. paniculata* demonstrating antiplasmodial activity as shown in Table 1, which has cytokine-modulating effects through the inhibition of GSK3β [67]. Nevertheless, other compounds may also contribute, or their interaction may eventually influence the extract activity. From these studies, the ethanolic extracts and ethyl acetate fraction might be investigated in a broader clinical study to confirm their efficacy for multi-compound product development, and andrographolide appears to be a definite bioactive compound, given its consistency in the in vitro and in vivo studies.

The acute toxicity study from the first true leaf ethanolic extract of *A. paniculata* in mice showed that a single oral administration with a dose of 5000 mg/Kg BW has no significant acute toxicological effect [110]. Another acute toxicity study showed that the methanolic extract of *A. paniculata* leaves at a dose of 5000 mg/Kg BW did not pose any treatment-related adverse clinical signs after 14 days of administration [111]. Moreover, the ethanolic extract of the aerial part of the plant did not show any testicular toxicity at a dose of 1000 mg/kg BW after 60 days of forced administration by buco-gastric formula [112]. In the acute toxicity study of its bioactive compound andrographolide, a dose of 500 mg/kg BW orally did not cause any toxicity; hence, it is considered relatively nontoxic [113]. Another study of doses up to 5 g/kg BW orally in mice did not show any signs of acute toxicity, whereas oral administration at a dose of up to 500 mg/Kg BW in Wistar rats for 21 days for subacute toxicity evaluation did not result in death or other signs of toxicity [114]. These studies suggest the relatively nontoxic properties of *A. paniculata* extracts and andrographolide as its bioactive compound.

## 6. *Physalis minima* L.

**Figure 8:**
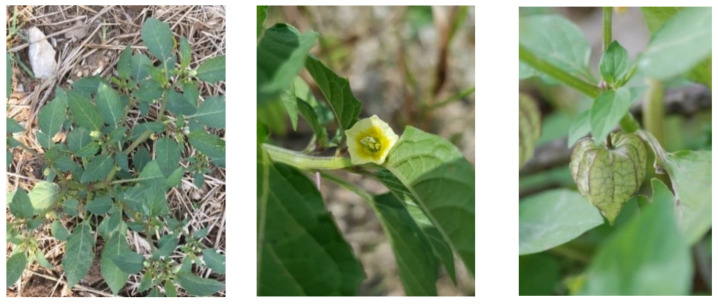
*Physalis minima* L. [119].

*Physalis minima* Linn. (Solanaceae) is a small herbaceous annual plant grown as a weed in crop fields, found widely in Indonesia, Malaysia, India, Afghanistan, tropical Africa, and Australia (Figure 8) [115,116,117,118]. The plant has a bitter taste and is known in Indonesia as ciplukan and, in Papua Island, it is locally known as pakpak, toki, daun nipon, cemploka, and seberi-nembai [14,117,118].

### 6.1. Ethnopharmacology

Traditionally, *P. minima* is used as an antiulcer, anti-inflammatory, antimalarial, antidiabetic, antimicrobial, diuretic, purgative, and cardioprotective plant [118,120]. The fruits and flowers are used to treat stomach pain and constipation. Herb paste is also applied to ear disorders [121,122]. In the Malay community, the decoction of the whole plant is used to cure cancer [123]. In Papua Island, Indonesia, *P. minima* is among the top four medicinal plants used to combat malaria; however, no specific usage was explained in the literature [14].

### 6.2. Phytochemistry

*P. minima* contains physalins A, B, D, F, H, I, L, and X, withaphysalins A, B, C, D, and E, and physalindicanols A and B [124,125,126,127]. Other studies reported that aerial parts of *P. minima* contain withaferin A, withanolide A, stigmasterol, sitosterol, withanone, dihydroxyphysalin B2-4, isophysalin B, withaminimin, 3-*O*-gylcoside of kaempferol and quercetin, and phygrin [126,128,129,130]. Another study reported the isolation of flavonoid compounds, namely, 5-methoxy-6,7-methylenedioxyflavone and 5,6,7-trimethoxyflavone [131].

### 6.3. Antiplasmodial Activity

**Figure 9:**
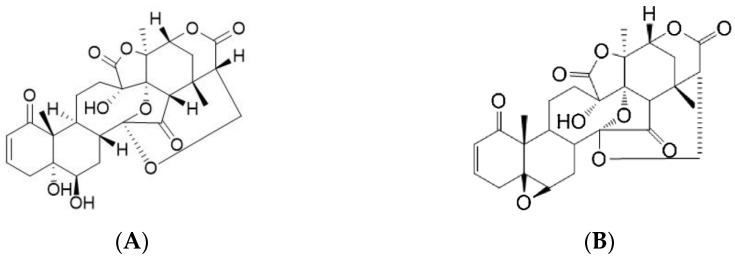
Structures of physalin D (**A**) and physalin F (**B**).

To date, *P. minima* is less studied among the four plants used as antiplasmodial treatments in Papua Island; in fact, we have not found an antiplasmodial or antimalarial study of the plant. However, in Indonesia, *P. minima* is known as “ciplukan” and another species, *Physalis angulate*, is also called “ciplukan”. Lusakibanza et al. (2010) investigated the in vitro and in vivo antiplasmodial activities of *P. angulata*, whose methanolic and choloroform extracts demonstrated a strong in vitro activity according to Ouattara et al. against the chloroquine-sensitive *P. falciparum* strain 3D7 with IC_50_ of 1.25 and 1.96 µg/mL, and against the chloroquine-resistant *P. falciparum* strain W2 with IC_50_ of 3.02 and 2.00 µg/mL, respectively. The in vivo study showed that the aqueous extract at a dose of 300 mg/kg BW achieved 58.7% parasite suppression which, according to Munoz et al., is considered good [48,132]. Moreover, Sa et al. (2011) investigated the antiplasmodial properties of physalines B, D, F, and G. These compounds are also present in *P. minima*. The in vitro study showed that physaline F (Figure 9B) is the most active, with the lowest IC_50_ of 2.2 µg/mL, and physaline D (Figure 9A) is the less active among them with IC_50_ of 55 µg/mL. Interestingly, in the in vivo study, physaline D showed better activity with 65% parasite suppression at a dose of 100 mg/kg BW, which was considered very good according to Munoz et al., compared with physalin F with no parasite suppression at 100 mg/kg BW. Thus, suppression induced by physalin F might inhibit the antimalarial immune response to control infection in infected animals (Table 1); therefore, the better antimalarial activity of physalin D is probably due to its lack of immunosuppressive activity in mice [48,69]. Physalin D inhibits the P2X7 receptor [133]. *Plasmodium* infection releases large amounts of ATP, which results in sustained P2X7 receptor activation leading to a pro-inflammatory cycle [134]. Thus, treatment with P2 inhibitor that blocks these receptors will impair invasion [135]. This report is a good example of where the strong IC_50_ value of the in vitro analysis might not directly reflect a good in vivo activity as a more complex system was involved in the in vivo study, emphasizing the importance of the in vivo study. Given the lack of activity studies, a preliminary activity screening of the extracts is suggested.

In line with its pharmacological activity, toxicological data from *P. minima* is also limited. The water extract of *P. minima* leaves did not show any acute signs of toxicity at a dose of 2000 mg/kg BW after 14 days of oral administration [136]. The subchronic toxicity of the aqueous extract at a dose of 450 mg/Kg BW after 90 days of oral administration did not show any signs of hematologic toxicity, hepatotoxicity, or renal toxicity [137]. The lack of toxicity data for other organic solvent extracts such as methanol or ethanol, and its possible bioactive compound, physalins, is a challenge to the development of potential antiplasmodial agents from *P. minima* and to providing the safety profile.

## 7. Conclusions

Among these four medicinal plants traditionally used to treat malaria from Papua Island, Indonesia, *A. paniculata* is the most extensively studied and is a potential medicinal plant for antiplasmodial development, having completed the in vitro, in vivo, phases 1 and 2 of clinical trials, and a toxicity profile. The extract and its bioactive compound, andrographolide, have the potential to be developed as an antiplasmodial agent whether as a multicompound or single-compound product. *A. scholaris* and *C. papaya* also have the potential to be further investigated for determining their bioactive compounds as both posed good potential for antiplasmodial activities in vivo and have extensive toxicity data; however, the considered bioactive compounds must be evaluated for their in vivo antiplasmodial activity. *P. minima* is a less studied medicinal plant among the others; nevertheless, it allows for more potential investigation as this plant is also used extensively in traditional remedies to treat malaria.

## Data Availability

No new data were created or analyzed in this study. Data sharing is not applicable to this article.
